# Stability analysis of cocoa clones for yield in the humid tropics of India

**DOI:** 10.3389/fpls.2026.1767516

**Published:** 2026-04-13

**Authors:** Alwala Kireeti, Gunna Ramanandam, Valluri Govardhan Rao, Boddepalli Neeraja, N. B. V. Chalapathi Rao, Elain Apshara, S. Sumitha, Augustine B. Jerard

**Affiliations:** 1 Department of Horticulture, Dr. YSR Horticultural University, Venkataramannagudem, Andhra Pradesh, India; 2 Horticultural Research Station, Ambajipeta, Andhra Pradesh, India; 3 Crop Improvement, Indian Council of Agricultural Research (ICAR)-Central Plantation Crops Research Institute, Kasaragod, India

**Keywords:** cocoa, yield stability, genotype by environment, Eberhart–Russell regression, AMMI, humid tropic

## Abstract

**Introduction:**

Climate change, marked by rising temperatures and shifting rainfall patterns, is known to impact cocoa growth thus fluctuating cocoa production in various countries. The objective of this study is to assess the yield stability and adaptability of different cocoa clones under varying environmental conditions and to identify genotypes suitable for climate-stressed and marginal environments.

**Methods:**

Six cocoa clones—VTLCC1, VTLCH1, VTLCH2, VTLCH3, VTLCH4, and VTLC1 (control) laid in RCBD with four replications as intercrops in a coconut plantation —were evaluated over four consecutive years (2020–2023) to assess variability in dry bean yield and related traits.

**Results:**

The combined regression, additive main effects and multiplicative interaction (AMMI), and variance-based stability analyses demonstrated a significant genotype-by-environment (G × E) interaction and clones differed widely in terms of dry bean yield and other yield parameters. VTLCH2 noted to be the most productive cocoa clone, exhibiting superior pod yield (41.36), higher bean number (41.90), dry bean yield (2.07kg/plant) and stable fat content across environments. Its β_i_ < 1, low s^2^d_i_, and favourable PC1 positioning indicated specific adaptability to marginal or stress-prone environments, making it suitable for humid tropics under climate-change conditions.

**Discussion:**

VTLCH2 consistently recorded superior performance across years that differed in rainfall and temperature, indicating better tolerance to seasonal fluctuations in moisture availability and higher temperature conditions compared with the other clones, as indicated by a negative PC1, and positive PC2 near to unity in AMMI, confirmed the suitability of cocoa clone VTLCH2 for marginal (unfavorable) climate.

## Introduction

Cacao (*Theobroma cacao* L.) is a perennial crop originated from the Spanish term “cacao.” The plant belongs to the family Malvaceae. Cocoa is native to the Amazon Basin (Motamayor et al., 2008) as a natural under-storey plant (Micheli et al., 2010), which requires specific climatic conditions for its development. Annual rainfall between 1,300 and 2,000 mm with a limited number of dry days (less than 3 months), an average daily temperature between 24°C and 28°C, and relative humidity between 80% and 90%, with daily sunshine duration of more than 4 h, are preferable (Chowdappa and Hubballi, 2018). Although not native to India, cocoa thrives in the humid tropics of the country. The maximum area of cultivation is confined to the peninsular states Andhra Pradesh, Kerala, Karnataka, and Tamil Nadu, cultivated as mixed crops or intercrops in coconut and oil palm orchards, covering an area of 1.03 lakh hectares with an annual yield of 27,702 metric tons (Anon., 2024). Despite this, India’s chocolate and confectionery sectors demand 50,000 metric tons of dry beans every year, necessitating imports valued at Rs 20,210 million due to domestic shortfalls.

Climate change, similarly to droughts and excessive rainfall, unfavorable weather conditions, affects the cocoa production in key growing regions such as Kerala, Tamil Nadu, and Andhra Pradesh, leading to lower yields and poorer quality beans, in turn creating huge fluctuations in the market price of dried cocoa beans (Asante et al., 2025). Investigations into the combined effects of climate change variables on cocoa are scarce. It is crucial to evaluate not only the responses of cocoa clones to individual climatic factors but also how the interactions of these variables affect the growth and productivity of cocoa. Harnessing the genetic diversity in cocoa by identifying high-yielding clones is essential for combating the impacts of climate variability on constant yields at various time periods at a location. The growing unpredictability of climate has diminished crop yield stability, making stability analysis increasingly important for farmers. Consequently, stability assessment across different environments can aid in the selection of superior genotypes.

Exploitation of the genetic diversity in cocoa by evaluating 50 cocoa clones under Kerala conditions (Elain Apshara et al., 2003) for stability of pod yield over years has facilitated the selection of high-yielding varieties suitable to the humid tropical regions in India, which is crucial to mitigating the number of cocoa clones to evaluate. Genotypic variations in the productivity, net photosynthesis, canopy structure, and biomass distribution have been identified (M’bo et al., 2016). In addition, temperature variations have been shown to differentially affect pod development and bean quality (Daymond and Hadley, 2008). Stability analysis has been applied on the quality traits of cocoa. Significant genotype × environment interactions (GEIs) for the fat and polyphenol contents have been reported across contrasting environments in Indonesia (Afandi et al., 2023). Furthermore, performance studies have been conducted and reported in India (Phukon et al., 2021; Sumitha et al., 2025). However, stability analyses in terms of GEI studies with additive main effects and multiplicative interaction (AMMI), the Eberhart and Russell (1966) model, and non-parametric stability analysis were explored in this article. Yield stability is defined by consistent agricultural outputs over time, across diverse environments and seasons, thereby ensuring reliability of varietal performance under fluctuating agro-climatic conditions (Seyed et al., 2025). Therefore, evaluation of the stability of various cocoa clones over a period helps in the identification of superior genotypes suitable for sustainable yields. Therefore, this study was conducted with the objective of evaluating and identifying superior cocoa clones that combine high yield potential with stability and resilience under stress conditions associated with variable annual conditions, such as elevated temperatures and irregular rainfall.

## Materials and methods

A field trial to evaluate the performance of cocoa clones as intercrops in coconut gardens was initiated in 2008 at the Dr. YSR Horticultural University (Dr. YSRHU) – Horticultural Research Station, Ambajipeta, Dr. B. R. Ambedkar Konaseema District. The experimental site is located in the east coast region of Andhra Pradesh at 16°59'38˝ N latitude, 81°56'37˝ E longitude, at an elevation of 14 m above mean sea level, under the ICAR–AICRP on Plantation Crops.

The trial was conducted on coastal alluvial soils with a clay loam to clay texture. The soil has a pH of 8.21, electrical conductivity (EC) of 0.10 dS m^−1^, organic carbon content of 0.6%, available nitrogen of 240 kg ha^−1^, phosphorus of 16.2 kg ha^−1^, and potassium of 220 kg ha^−1^. The annual rainfall during the experimental period was 1,459.7 mm, while the minimum and maximum temperatures ranged from 23.4°C to 24.7°C and from 31.1°C to 36.3°C, respectively (**Table 1**). The site falls under the Krishna–Godavari zone, which is characterized by a tropical humid climate with distinct summer, winter, and rainy seasons. The average relative humidity varies between 65.7% and 87.2%, as recorded by an automatic weather station installed at the study site by the India Meteorological Department.

**Table 1:** Environmental characteristics of the experimental site related to the evaluation of cocoa clones.

Site	Geographical position	Year	Temperature (°C)		RH (%)		Annual rainfall (mm)	No. of rainy days
			Maximum	Minimum	Maximum	Minimum		
Dr.YSRHU – Horticultural Research Station, Ambajipeta, Andhra Pradesh, India	16°59'38˝ N latitude, 81°56'37˝ E longitude, 14 m.a.s.l.	2019	32.7	23.4	88.61	60.19	824.7	47
		2020	31.7	24.5	82.66	65.87	3321.6	78
		2021	31.1	24.7	86.99	66.57	1406.5	71
		2022	34.7	23.7	87.00	66.49	1039.5	71
		2023	36.3	23.4	84.94	56.21	704.6	73

*RH*, relative humidity; mm, milli meter; m.a.s.l, metres above sea level;N latitude, North latitude; E longitude, East longitude; °C, centigrade.

The experiment was laid out in a 27-year-old coconut orchard planted with the hybrid Godavari Ganga (East Coast Tall × Ganga Bondam Green Dwarf), an inter-varietal cross developed for early flowering and higher yields. Six cocoa clones were considered as six treatments and were evaluated using a randomized complete block design (RCBD) with four replications in an area of 0.8 ha. Each treatment consisted of six cocoa plants per replication. The six cocoa clones evaluated were VTLCC1, VTLCH1, VTLCH2, VTLCH3, VTLCH4, and VTLC1 (control) (**Table 2**). Cocoa was planted as a single-row intercrop between adjacent coconut rows planted at 7.5 m × 7.5 m. Cacao was planted at a spacing of 3.75 m from the coconut trunk and with 3.0 m between cacao plants, resulting in a planting density of 244 cacao plants ha^−1^. All recommended agronomic practices were followed uniformly throughout the experimental period. 

**Table 2:** Details of the cocoa clones/hybrids under evaluation.

Cocoa clone	Parentage
Vittal cocoa clone-1 (VTLCC1)	Selection from Nigerian germplasm
Vittal cocoa hybrid-1 (VTLCH1)	Hybrid developed by crossing Malaysian collections (EC 631540 × EC 631534)
Vittal cocoa hybrid-2 (VTLCH2)	Hybrid developed by crossing Lalbaugh collections (IC 565551 × IC 565556)
Vittal cocoa hybrid-3 (VTLCH3)	Hybrid developed by crossing Malaysian and Nigerian collections (EC 631534 × EC 631546)
Vittal cocoa hybrid-4 (VTLCH4)	Hybrid developed by crossing Malaysian and Nigerian collections (EC 631534 × EC 631556)
Vittal cocoa selection-1 (VTLC1)	Selection from indigenous germplasm (IC0597837)

Pruning was carried out regularly, wherein chupons arising from the main stem and fan shoots were removed before and after each monsoon. Dried and diseased branches were also removed periodically. Training of cacao trees was practiced by removing approximately 20% of vegetative growth annually during July and August to facilitate light penetration, minimize pest and disease incidence, and create a favorable microclimate for enhanced flowering. All cacao plants were flood-irrigated during the study period.

Biometric observations, including plant height, trunk girth, jorquetting height, canopy spread (east–west and north–south), total canopy area, single fruit weight, number of fruits per tree, single bean weight, dry bean characteristics, and yield, were recorded and analyzed. For dry bean analysis, the harvested cocoa pods were stored under shade, and wet beans were fermented for 6–7 days in wooden baskets. Following fermentation, the beans were sun-dried naturally for 7–8 days at approximately 30°C to obtain well-dried cocoa beans.

The cacao plants under evaluation were monitored regularly, and standard management practices recommended by Dr. YSRHU–HRS, Ambajipeta, were followed throughout the experimental period. Data on the vegetative and yield parameters were recorded for each plant separately. Mean values were calculated from six plants per replication. Vegetative parameters such as plant height (in meters), stem girth (in centimeters), canopy spread, and height at first branching (in meters) were recorded during 2023–2024, when the plants were 15 years old. Yield-related traits, including the number of pods per plant, the number of beans per pod, the dry bean yield per plant, and the fat content of the cocoa beans, which were recorded over 4 years (2020–2023), were used for the present study. Vegetative characterization was carried out following the standard procedures prescribed by the International Union for the Protection of New Varieties of Plants (UPOV).

### Stability analysis

#### Eberhart and Russell (1966) model of regression-based stability analysis

Stability analysis was performed using the Eberhart and Russell model. In this model, the environmental index was calculated by subtracting the grand mean from the mean yield of all genotypes in each environment. In addition to the regression coefficient (*β_i_*), the mean square deviation from regression (*S*²*d_i_*) was estimated. The deviation from regression measures the departure of an individual genotype from its linear response to environmental changes. A highly stable genotype is characterized by *β_i_* = 1.0 and *S*²*d_i_* = 0. Genotypes with *β_i_* > 1 are considered better adapted to favorable environments, while those with *β_i_*< 1 are considered better adapted to marginal or unfavorable environments. This method has been widely used in stability studies of horticultural and field crops.

Mathematically, the model is expressed as:


$$Y_{ij}=\mu_{i}+\beta_{i}I_{j}+\delta_{ij}$$

where *Y_i_* is the mean performance of the *i*th genotype in the *j*th environment; *μ_i_* is the mean of the *i*th genotype across environments; *β_i_* is the regression coefficient representing the response of the genotype to environmental changes; *I* is the environmental index; and *δ_i_* is the deviation from regression (nonlinear component).

GEIs were examined using stability analysis, referring to the conventional variance (CV) by Shukla (1972). Wricke (1962) mentioned that equivalence (*W_i_*^2^), as a stability parameter, measures the contribution of each clone to the square of the total interaction between the clone and the environment. The formula is: x-


$$W_{i}^{2}=\sum_{j=i}^{a}{\left(x_{ij\ldots }-\bar{x_{j}}+\ldots \right)}^{2}$$

#### Non-parametric stability analysis

In this model of stability analysis, the genotypes are considered stable if the ranks for each environment are similar across environments in each statistic proposed by Thennarasu (1995), which employs non-parametric stability indices [NP*_i_*^(1)^, NP*_i_*^(2)^, NP*_i_*^(3)^, and NP*_i_*^(4)^] as stability parameters. The value of the non-parametric stability index is obtained from the corrected average ranks of the genotypes. Furthermore, non-parametric stability indices based on rankings of R_a (superiority index for all environments), R_f (superiority index for favorable environments), and R_u (superiority index for unfavorable environments) were calculated using a regression model, Lin and Binns’ (1988) method, Shukla’s variance, and Wricke’s ecovalence. Higher stability was considered with the lowest ranking across the various genotypes with similar rankings across the various stability indices.

### Additive main effects and multiplicative interaction analysis for genotype × environment interactions

The AMMI statistical technique is used in plant breeding to evaluate and interpret GEIs under the assumption that the errors are normally distributed. The AMMI stages include: 1) compiling a matrix of the interaction effects of genotypes and locations; 2) performing a bilinear breakdown of the matrix through singular value decomposition (SVD); 3) determining the number of principal fundamental components through postdictive success; and 4) constructing AMMI biplots (Sujiprihati et al., 2006). AMMI has become a popular statistical tool among agricultural researchers for the purpose of understanding GEIs and gaining accuracy in the selection of stable genotypes in many crops, such as wheat (Singh et al., 2020) and oilseed rape (Agahi et al., 2020).

The data recorded over 4 years were initially subjected to two-way analysis of variance (ANOVA) considering genotypes and years as factors for the GEI analysis. The ANOVA for RCBD was conducted using OPSTAT, and means were compared at the 5% significance level. All other statistical analyses were performed using R software (version 4.4.2.) with the packages agricolae, metan, AMMIdesign, ggplot2, and dplyr. The stability parameters were estimated following Eberhart and Russell (1966). The significance of the regression coefficient (*β_i_*) was assessed using the *F*-test, and the deviation from regression (*S*²*d_i_*) was assessed for significance using appropriate variance tests. AMMI analysis was conducted using the metan package, and biplots were generated using ggplot2.

## Results

Data pertaining to the growth characteristics in the experiment on the performance of cocoa clones as intercrops, including girth, height at first branching, and canopy spread for the year 2023, recorded no significant differences as the trees were trained and pruned regularly, which is the recommended practice in cocoa cultivation. The plant height ranged between 3.03 m (VTLCH3) and 3.39 m (VTLC1). The east–west (E–W) spread was lowest in VTLCH2, with 3.68 m, and maximum in VTLCH3, with 4.12 m, while the north–south (N–S) spread was also recorded to be greater in VTLCH3 (3.61 m) and lower in VTLCH4 (3.24 m). Although the data on the growth parameters were not statistically significant, there may be some significant impact of these growth parameters on the yield attributes. The uniformity of the vegetative traits studied supports the interpretation that yield variations are primarily due to reproductive traits and GEI rather than vegetative growth alone.

The average dry bean yield was found maximum in VTLCH2, with 2.10 kg plant**^−^**^1^ year**^−^**^1^, which was significantly on par with that in VTLCH4 (1.80 kg plant ^−1^ year^−1^) and superior over those of the other cocoa clones evaluated. For the number of pods per plant, the cocoa clone VTLCH2 was recorded to produce the maximum number, with 41.36 pods, which was on par with those of VTLCH4 and VTLCH1, with 38.81 and 36.80 pods per plant per year, respectively. The number of beans per pod was noted to be greater in VTLCH2 (41.90) and significantly on par with those of VTLCH4 (40.17) and VTLCH3 (38.25). The cocoa clone VTLC1 showed lower performance for dry bean yield, number of pods per plant, and number of beans per pod, with 1.41 kg plant**^−^**^1^ year**^−^**^1^, 35.49, and 35.43, respectively. Quality traits including the fat content in the cocoa bean were found to be non-significant among the cocoa clones evaluated.

The environment (year)-wise performance of cocoa for dry bean yield, number of pods per plant, and number of beans per pod showed significant variations among the years (**Table 3**). The dry bean yield ranged from 1.48 to 1.75 kg plant^−1^ during the study period. The highest yield was recorded in 2023 (1.75 kg plant^−1^), significantly on par with those of 2020 (1.70 kg plant^−1^) and 2022 (1.66 kg plant^−1^), while the lowest yield was noted in 2021 (1.48 kg plant^−1^). The maximum number of pods was recorded in 2020 (42.61) and was significantly superior over those of other years, whereas the minimum was observed in 2023 (32.48). The highest number of beans per pod was observed in 2021 (41.24) and was significantly superior over those of other years, while 2020 and 2023 recorded the lowest value (35.78). While the environment (year) × treatment interaction effect was significant only for dry bean yield, indicating that varietal performance differed across years for this trait, for the other measured parameters, the interaction was not significant.

**Table 3:** Performance of the cocoa clones for the yield parameters from 2020 to 2023.

Cocoa clone/Year	Dry bean yield (kg plant^−1^)	No. of pods plant^−1^	No. of beans pod^−1^
Cocoa clone			
VTLCC1	1.48	35.73	36.19
VTLCH1	1.50	36.80	36.00
VTLCH2	2.10	41.36	41.90
VTLCH3	1.60	35.42	38.25
VTLCH4	1.80	38.81	40.17
VTLC1	1.41	35.49	35.43
±SEM	0.07	1.32	0.89
CD at 5%	0.20	3.76	2.53
CV (%)	16.21	14.39	9.35
Year			
2020	1.70	42.61	35.78
2021	1.48	38.36	41.24
2022	1.66	35.62	39.16
2023	1.75	32.48	35.78
±SEM	0.05	1.09	0.73
CD at 5%	0.16	3.08	2.06
CV (%)	17.15	14.3	9.4

*CD*, critical difference; *CV*, coefficient of variation.

**Figure 1:**
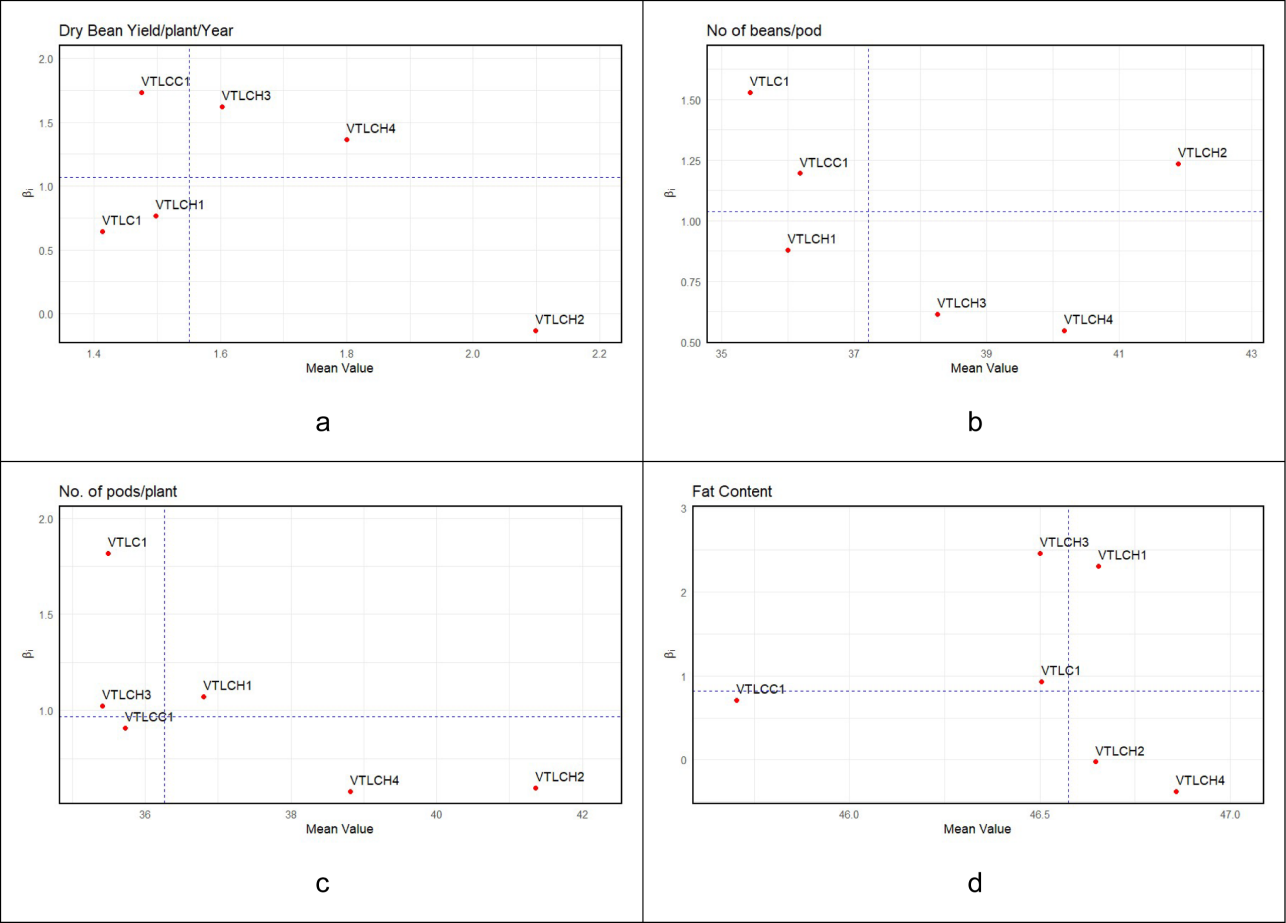
**(a–d)** Graphs depicting the stability parameter (mean, regression coefficient (*β_i_*)) for dry bean yield per plant per year **(a)**; for the number of beans per pods **(b)**; for the number of pods per plant per year **(c)**; and for fat content **(d)**.

The Eberhart and Russell regression-based stability analysis revealed differential responses of the cocoa clones across environments for dry bean yield, number of pods per plant, number of beans per pod, and the fat content traits (**Figure 1**). For dry bean yield, the regression coefficients (*β_i_*) ranged from −0.13 to 1.73, indicating variable adaptability among genotypes. Clone VTLCC1 (*β_i_* > 1) showed high responsiveness to favorable environments, followed by VTLCH3. Those of VTLCH1, VTLC1, and VTLCH4 were near 1 (*β_i_* ≈ 1), exhibiting average stability and consistent yield across locations, representing that the yields were consistent and less influenced by environmental fluctuations. VTLCH4 recorded a higher dry bean yield compared with the overall mean for dry bean yield among the three cocoa clones. VTLCH2 (*β_i_*< 1) performed better than the other clones under evaluation in marginal (unfavorable) environment conditions, followed by VTLCH4 (*β_i_* > 1), which performed well under productive (favorable) environments. From the regression plot in **Figure 1a**, **Table 2**, it can be observed that VTLCH4 was stable, with 1.8 kg of dry bean yield per plant per year and with *β_i_* (1.36) almost near to 1.

For another important yield trait, i.e., the number of pods per plant, the majority of the clones showed *β_i_* values near unity (**Figure 1c**), suggesting general adaptability of these cocoa clones across the various years. VTLC1 had a *β_i_* > 1, indicating superior response under favorable growing conditions, whereas VTLCH4 and VTLCH 2 (*β_i_*< 1) were more suited to marginal (unfavorable) environments. Furthermore, these were positioned far away from the median line for the mean dry bean yield, depicting superiority over the other clones evaluated. Their proximity to *β_i_* indicates that the pod production in these clones was relatively less stable, suggesting minimal environmental influence compared with VTLC1. Therefore, VTLCH4, with 38.81, and VTLCH2, with 41.36, were considered as reliable performers under both favorable and suboptimal conditions for number of pods per plant.

For the number of beans per pod in **Figure 1b**, the regression coefficients revealed moderate variations among genotypes. VTLCH2 and VTLCC1 exhibited *β_i_* > 1 and higher mean values, reflecting strong performance in favorable environments, while VTLCH3 and VTLCH4 (*β_i_*< 1) were relatively stable, but less responsive. The cocoa clone VTLCH1 had *β_i_* values around 1, indicating stable bean setting ability across environments, but with fewer beans per pod for the cocoa clones evaluated. The cocoa clone VTLCH2 exhibited more number of beans per pod, with 41.90, and near to unity for *β_i_*, indicating consistent bean setting and bean counts, and performed well under favorable environments, which is a desirable attribute for yield stability in cocoa.

In the case of fat content (**Figure 1d**), genotypes VTLCH1 and VTLCH3 had *β_i_* > 1 with higher mean values, suggesting better performance in favorable environments. VTLCH2 and VTLCH4, with *β_i_*< 1, were stable under average or suboptimal conditions. **Table 2**, **Figure 1d** show that all of the cocoa clones evaluated performed similarly in terms of fat content over the years, except for the cocoa clone VTLCC1. This stability is valuable for quality consistency in cocoa processing. The highest fat content was recorded in VTLCH4, with 46.86, followed by VTLCH2 and VTLCH1, with 46.65.

The regression-based stability analysis following Eberhart and Russell (1966) revealed the differential adaptability of the cocoa clones across environments for yield and quality traits. VTLCH2 (*β_i_*< 1) exhibited superior and consistent performance under marginal and suboptimal environments, showing stability for dry bean yield (2.10 kg plant^−1^ year^−1^), number of pods per plant (41.36), number of beans per pod (41.90), and fat content (46.65%). VTLCH1 and VTLCH3 (with *β_i_* near to unity) were characterized by high stability and broad adaptability across environments, but with low yield traits compared with the other cocoa clones evaluated, whereas VTLCC1 and VTLCH4 (*β_i_* > 1) showed better responses under favorable conditions. Overall, the results confirmed VTLCH2 as the most consistent and promising clone for sustained productivity and quality across variable growing environments.

**Figure 2:**
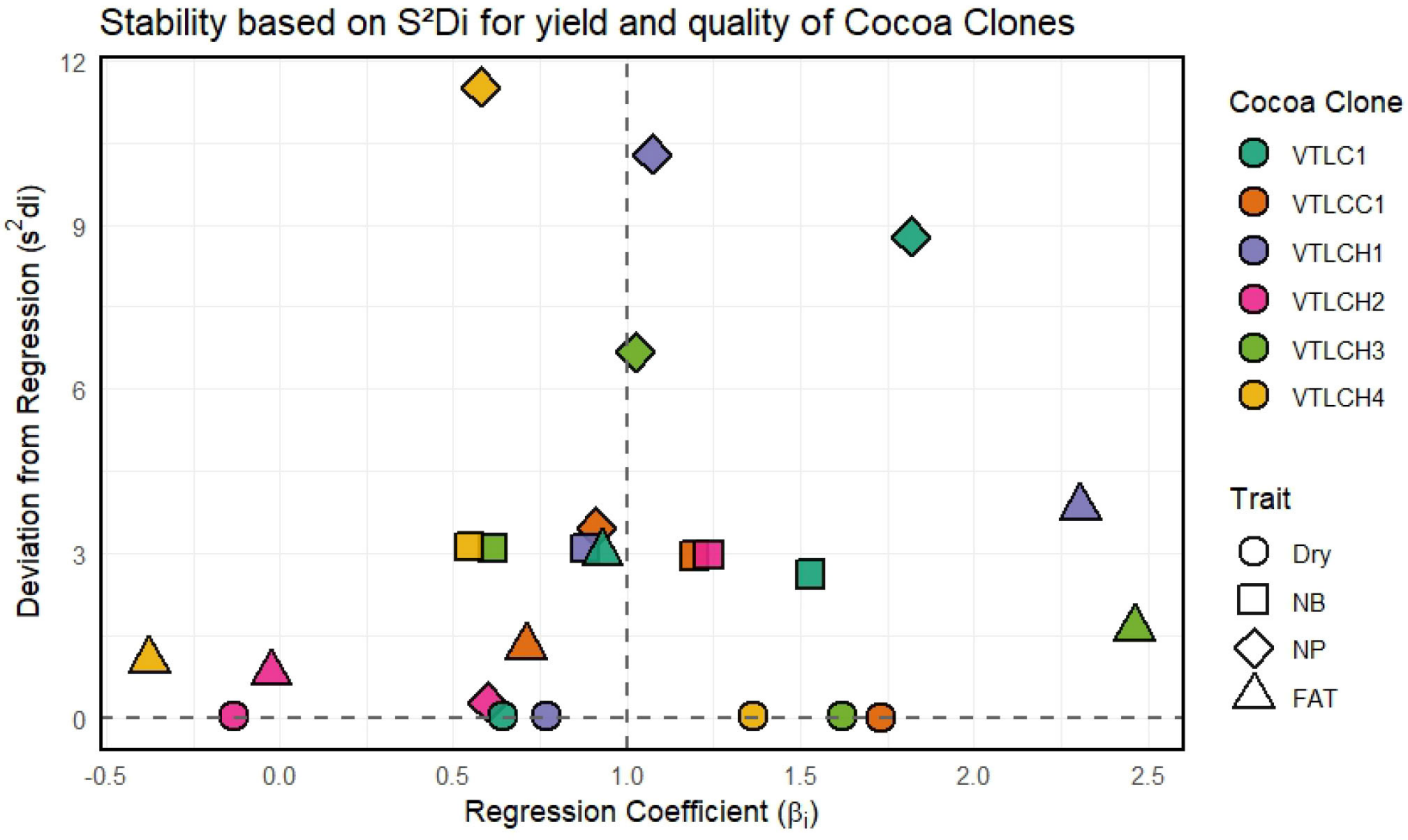
Stability based on the deviation from regression (*S*^2^*d_i_*) *vs*. the regression coefficient (*β_i_*).

The stability analysis based on the deviation from regression (*S*^2^*d_i_*) and the regression coefficient (*β_i_*) in Eberhart and Russell’s regression stability model revealed distinct adaptability patterns across environments (**Figure 2**). For dry bean yield, all of the cocoa clones evaluated were near to zero for *S*^2^*d_i_*, which explains that the yield of the cocoa clones can be predicted with the regression model as the deviation from regression was low.

Among the various yield traits evaluated, the *S*^2^*d_i_* values of the fat contents in VTLCH2, VTLCH4, VTLCC1, and VTLCH3 were near to zero, with negative regression coefficients (*β_i_*) confirming the lower performance of these cocoa clones under marginal (unfavorable) environment conditions. On the other hand, for the number of pods per plant, the *S*^2^*d_i_* of VTLCH2 was close to zero, indicating that the performance of this clone for the above trait was consistent and predictable as the deviation from regression was low and close to unity for the regression coefficients (*β_i_*), confirming that the performance of cocoa clone VTLCH2 was stable across environments (**Figure 2**). The other yield traits, including the number of beans per pod and the number of pods per plant, were unpredictable as the *S*^2^*d_i_* values were high.

These findings highlight that the cocoa clones, for the dry bean yield and fat content traits, were predictable using the Eberhart and Russell model of regression stability analysis. Based on both **Figures 1**, **2**, it was confirmed that the cocoa clones exhibited significant G × E variations for the above yield traits, and based on the yield results in **Table 3**, as well as **Figures 1**, **2**, genotypes VTLCH4 (1.80 kg plant^−1^ year^−1^) and VTLCH2 (2.10 kg plant**^−^**^1^ year**^−^**^1^) showed high performance and good adaptability in marginal (unfavorable) environments, such as heavy rainfall or high maximum temperature. 

*W_i_*^2^, Ecov_perc, and *σ*^2^*_i_* denote Wricke’s ecovalence, the ecovalence percentage, and Shukla’s variance, respectively. VTLCH1 was observed to be stable compared with the other clones evaluated, with Wricke’s ecovalence (*W_i_*^2^), ecovalence percentage (Ecov_perc), and Shukla’s variance (*σ*^2^*_i_*) of 0.02, 1.56%, and −0.002, respectively, for consistent dry bean yield and 12.30, 2.39%, and −0.61, respectively, for the production of pods per plant per year (**Table 4**). The *W_i_*^2^ (1.82), Ecov_perc (2.47%), and *σ*^2^*_i_* (−0.08) values of VTLCH1 also confirmed this clone as the most stable and suitable for consistent number of beans per pod compared with the other clones evaluated. For fat content, VTLCH2, with 4.64, 3.53%, and 0.03, followed by VTLCH4, with 4.70, 3.57%, and 0.04, were observed to have the lowest *W_i_*^2^, Ecov_perc, and *σ*^2^*_i_*, respectively. Therefore, for fat content, VTLCH2 was found stable over the other clones, while VTLCH1 was found stable for the other yield parameters such as dry bean yield, with a moderate yield of 1.50 kg plant^−1^ year^−1^.

**Table 4:** Genotype-by-environment interactions based on Wricke’s ecovalence, the ecovalence percentage, and Shukla’s variance for the cocoa clones studied.

Genotype	Dry bean yield plant^−1^ year^−1^			No. of pods plant^−1^			No. of beans pod^−1^			Fat content		
	*W_i_* ^2^	Ecov_perc (%)	*σ* ^2^ *_i_*	*W_i_* ^2^	Ecov_perc (%)	*σ* ^2^ *_i_*	*W_i_* ^2^	Ecov_perc (%)	*σ* ^2^ *_i_*	*W_i_* ^2^	Ecov_perc	*σ* ^2^ *_i_*
VTLCC1	0.18	17.27	0.018	122.95	23.91	13.23	4.92	6.69	0.31	19.28	14.64	1.86
VTLCH1	0.02	1.56	−0.002	12.30	2.39	−0.61	1.82	2.47	−0.08	43.46	33.00	4.88
VTLCH2	0.26	25.01	0.028	127.10	24.72	13.75	6.34	8.62	0.49	4.64	3.53	0.03
VTLCH3	0.08	8.17	0.006	40.09	7.80	2.87	13.49	18.35	1.38	26.76	20.32	2.80
VTLCH4	0.42	41.23	0.048	40.52	7.88	2.92	18.24	24.80	1.97	4.70	3.57	0.04
VTLC1	0.07	6.76	0.004	171.27	33.31	19.27	28.74	39.07	3.29	32.86	24.95	3.56

**Figure 3:**
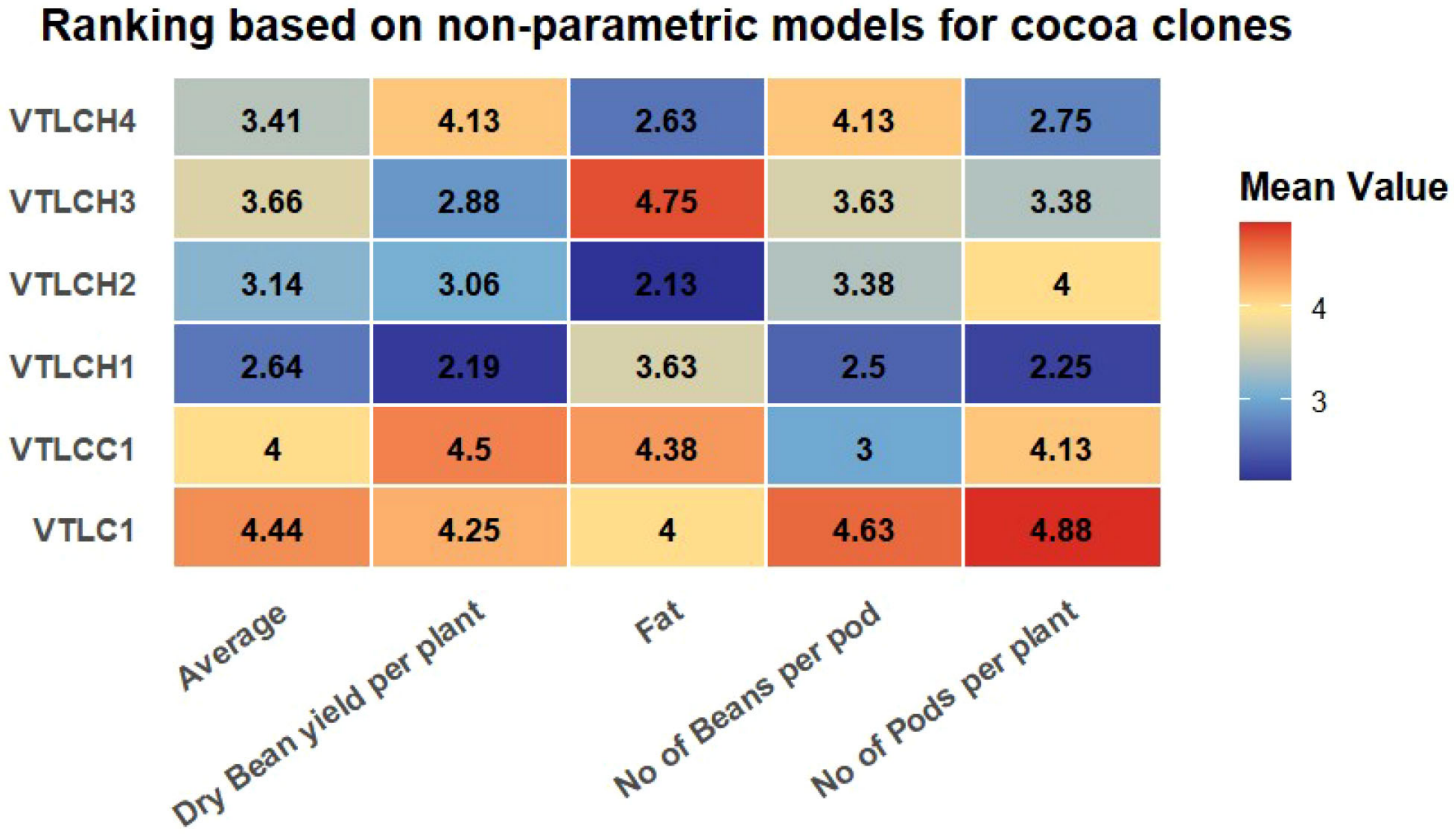
Heat map of the ranking performance of the cocoa clones across various yield traits.

The average rankings from various non-parametric models, such as Lin and Binns (R_a, R_f, and R_u), Shukla’s variance (*R σ*^2^*_i_*), and Thennarasu [NP^(1)^, NP^(2)^, NP^(3)^, and NP^(4)^], were averaged and are presented in **Figure 3**. The heat map of the ranking performance for the various traits and cocoa clones recorded VTLCH1 as stable, with the lowest average ranks of 2.19, 2.5, and 2.25 for the yield traits dry bean yield per plant in cocoa (in kilograms), number of beans per pod, and number of pods per plant, respectively, followed by VTLCH3 with 2.88 for dry bean yield per plant in cocoa, VTLCC1 with an average rank 3 for number of beans per pod, and VTLCH4 with 2.75 for number of pods per plant. On the other hand, VTLCH2 recorded the lowest with 2.13, followed by VTLCH4 with 2.63, and expressed stability for fat content over the other cocoa varieties or hybrids evaluated. The results for the non-parametric stability ranking index showed VTLCH1 to be stable, with minimum rank of 2.64 for all yield traits studied, followed by VTLCH2 with 3.14. From these results, it was determined that VTLCH2 may perform well in yield traits such as dry bean yield, number of pods per plant, and number of beans per pod, which were maximum in this clone with good stability compared with the other cocoa clones evaluated. 

Proportion and accumulated for PC1: 52.8, 52.8; PC2: 45.9, 98.7; and PC3: 1.3, 100.0

From the ANOVA (**Table 5**), it is clear that the perennial cocoa crop recommended for cultivation had a significant GEI and that the clones differed widely in their mean yield potential. Therefore, the selection of cocoa clones suitable for this region may focus on those with the highest overall mean yield.

**Table 5:** ANOVA of the additive main effects and multiplicative interaction (AMMI) analysis for dry bean yield.

Source	*df*	Sum of squares	Mean square	*F*-value	Pr(>*F*)
ENV	3	1.0207	0.3402	2.866	0.0809
REP(ENV)	12	1.4245	0.1187	1.662	0.0988
GEN	5	5.3647	1.0729	15.023	0.0000000015
GEN: ENV	15	2.0456	0.1363	1.905	0.039
PC1	7	0.5399	0.0771	1.080	0.387
PC2	5	0.4692	0.0938	1.310	0.272
PC3	3	0.0134	0.0044	0.060	0.981
Residuals	60	4.2851	0.0714	NA	NA
Total	110	15.1631	0.13784	NA	NA

ANOVA, Analysis of Variance; AMMI, Additive Main Effects and Multiplicative Interaction; ENV, Environment; REP (ENV), Replication within environment; GEN, Genotype; GEN × ENV, Genotype × Environment interaction; PC1, PC2, PC3, principal component axes; df, degrees of freedom; MS, mean square; Pr(>F), probability value.

### AMMI analysis for dry bean yield

**Figure 4:**
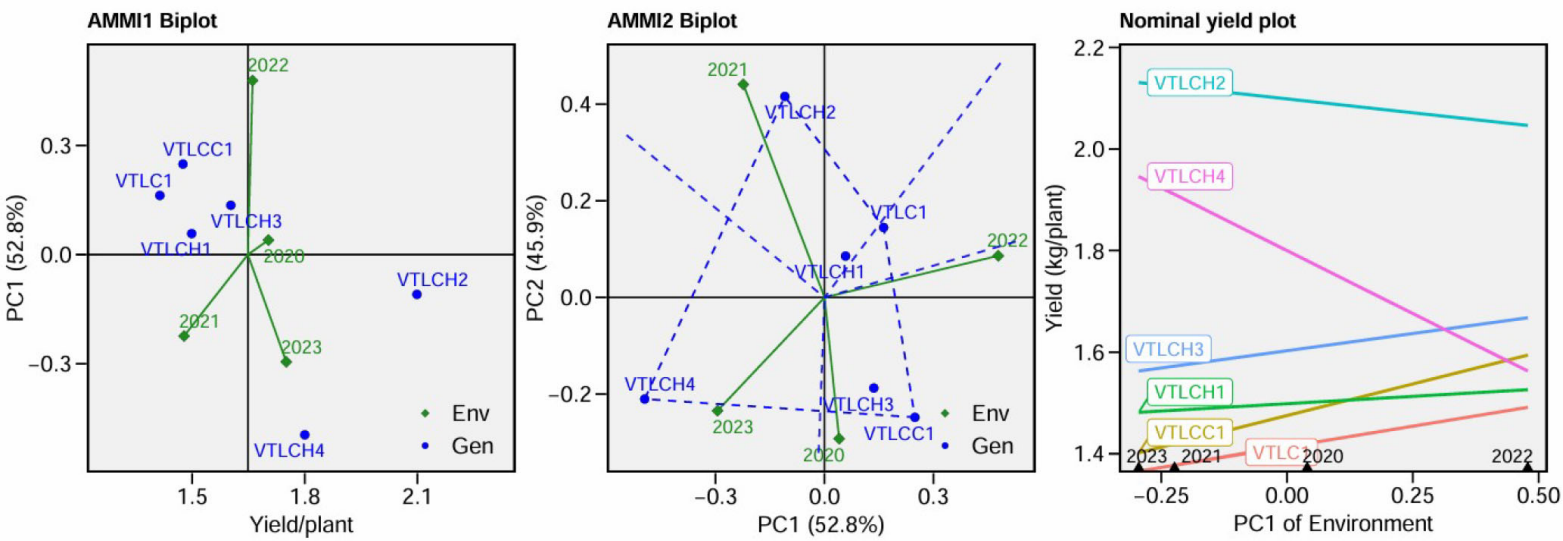
Additive main effects and multiplicative interaction (AMMI)-1 biplot; yield/plant *vs*. PC1, AMMI-2 biplot PC1 *vs*. PC2, and nominal yield plot.

The AMMI-1 biplot (**Figure 4**) revealed clear GEIs for dry bean yield. The AMMI analysis performed for the dry bean yield of the six cocoa clones showed that VTLCH1, VTLCH3, and VTLCH2 were the most stable as their PC1 values were close to zero. VTLCH2 showed potential for high yield in unfavorable environments as it was located in the negative quadrant of the PC1 axis, while the cocoa clones VTLCH1, VTLCH3, VTLC1, and VTLCC1 were found to be better for their overall performance across favorable conditions as they were located on the positive quadrant of PC1. VTLCC1 and VTLCH4 showed relatively larger PC1 scores, indicating stronger interaction with environments and reduced stability. These genotypes performed better in specific favorable environments, but were less consistent under various environments. The environment vectors showed that 2022 was strongly associated with higher yield performance, while 2020 and 2023 grouped more closely together, indicating similarity in yield performance between these years (**Figure 4**, AMMI-1 plot).

The **Figure 4**, AMMI-2 biplot showed that the most stable clone for dry bean yield is VTLCH1 as it was located closest to the origin of the biplot, indicating that it was less influenced by environmental variations and exhibited consistent performance across different environments. PC1 and PC2 explained major variation patterns for the dry bean yields of the cocoa clones studied as both explained 95.75% of the variation. On the other hand, VTLCH2 was recorded as the best clone well suited to favorable environments as it performed well under optimal conditions. VTLCH2 was located in the negative quadrant of the PC1 axis near to unity and positive to PC2, showing a pronounced GEI and performing particularly well in certain years/environments where this interaction was favorable.

PC1 often represents the main effect of environment. A positive PC1 value suggests that the genotype performs better under favorable conditions. For moderate PC2, the position of the genotype on the PC2 axis indicates its sensitivity to environmental interactions. A moderate PC2 value suggests that the genotype is moderately responsive to environmental changes, which can be beneficial in favorable conditions.

The **Figure 4**, Nominal yield plot clearly showed that VTLCH2 had the highest average dry bean yield per plant over the other cocoa clones in all environments, but performed well in unfavorable environments in 2020 and 2023 as the PC1 values for these years were negative, while VTLCH4 showed a decreased trend for yield with a steep slope from unfavorable environments to favorable environments. This suggests that it performed well across unfavorable environments. VTLCH1 was recorded to have an average yield compared with the other cocoa clones evaluated, and the moderate slope suggests that it was insensitive to environmental variations. Thus, VTLCH2 can be preferred for cultivation as it was recorded to produce approximately 2.1 kg of dry bean yield per plant, which was more than the general mean of 1.6 kg plant^−1^ and close to unity for the PC1 values.

**Figure 5:**
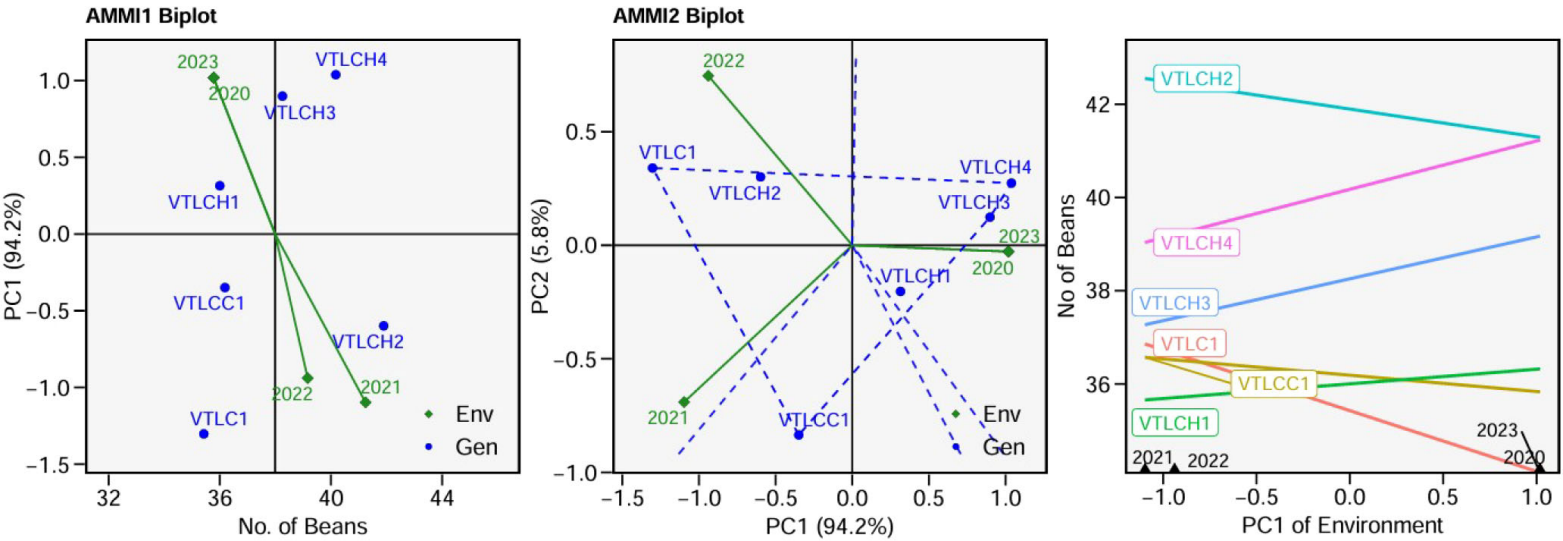
Additive main effects and multiplicative interaction (AMMI)-1 biplot for the number of beans *vs*. PC1, AMMI-2 biplot PC1 *vs*. PC2, and nominal bean yield per pod ofsix cocoa clones in various years.

The AMMI analysis performed for the number of beans per pod depicted that PC1 alone contributed 94.2% of the interaction diversity. Thus, it can be noted that the trait number of beans per pod was strongly influenced by genotype potential compared with environmental factor. VTLCH1 appears to be the most stable genotype as it was located closest to the origin, indicating minimal influence from environmental variations. VTLCH2 was recorded as suitable in a favorable climate for number of beans per pod. From the AMMI-2 biplot and the nominal biplot, VTLCH3 and VTLCH4 produced an increased number of beans per pod in favorable environments in 2020 and 2023 compared with the general mean of 38 beans per pod. From **Figure 5**, it can be derived that the performance for number of beans per pod was less affected by environmental variations, as captured by PC1.

**Figure 6:**
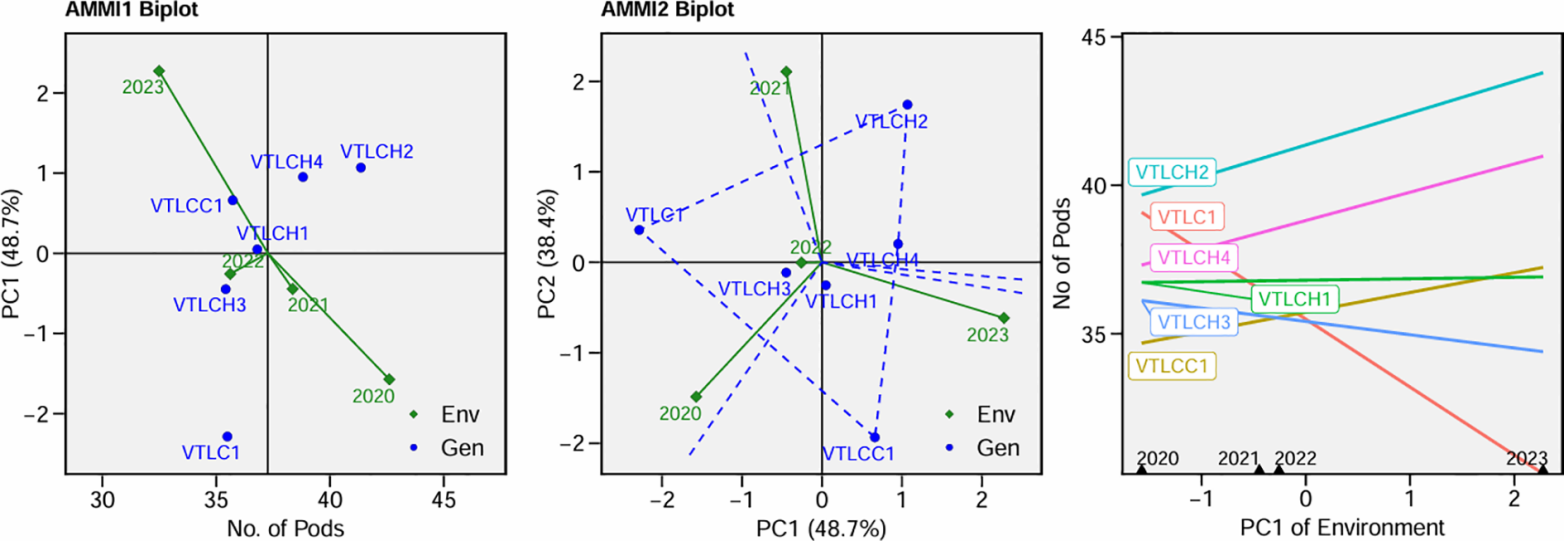
Additive main effects and multiplicative interaction (AMMI)-1 biplot for number of pods *vs*. PC1, AMMI-2 biplot PC1 *vs*. PC2, and nominal pod yield of six cocoa clones in various years.

The AMMI analysis for the number of pods per plant (**Figure 6**) noted stability in VTLCH1 and VTLCH3. The VTLCH2 and VTLCH4 genotypes performed best, showing the highest pod number, and produced more mainly in the 2023 environment. The environment in 2023 exhibited the highest PC1 scores, suggesting a favorable season, with high temperature favoring pod production. In the AMMI-2 biplot in **Figure 6**, two principal components together accounted for the majority (87.1%) of the patterns and variations in the number of pods per plant for the different cocoa clones and environments in this study. VTLCH1 and VTLCH3 showed fewer interactions, as indicated by the shorter vectors, confirming consistent pod numbers across environments. The cocoa clones VTLCC1, VTLCH4, and VTLCH 2 recorded higher number of pods over the years, suggesting good plasticity and adaptability; thus, these genotypes not only maintained but also improved their productivity as conditions changed—likely benefiting from both genetic potential and environmental factors. The negative PC1 values for the year 2020 showed that the 2019 environment was not favorable for number of pods per plant, while the positive PC1 value in 2023 may represent better conditions such as good rainfall during 2022, which might have favored pod development in 2023 and irrigation. These results provided a clear basis for recommending VTLCH2 and VTLCH4 for high pod yield in the experiment.

## Discussion

The superior performance of VTLCH2 was attributed to its genetic constitution that enhances reproductive efficiency and yield stability under variable environmental conditions. In cocoa, yield may be governed by the interactions of pod production number, bean setting efficiency, and assimilate partitioning, all of which were under strong genetic control (Elain, 2017; Mustiga et al., 2018). The inherent advantage of VTLCH2 appeared to arise from the coordinated regulation of these yield-determining traits, allowing more effective conversion of reproductive potential into economic yield.

Genotypic differences in cocoa clones have been known to influence pod initiation, fertilization success, and internal pod development through unique gene combinations that regulate flower retention, embryo development, and bean growth (Elain, 2017; Bhavishya et al., 2024). The superiority of VTLCH2 suggested an enhanced sink strength, which enabled efficient carbohydrate allocation to the developing beans and supported sustained yield formation under both optimal and stress-prone environments (Daymond and Hadley, 2008). Environmental sensitivity in cocoa is strongly linked to its cauliflorous flowering habit, where the flowering intensity and pod retention are tightly regulated by climatic factors such as temperature and rainfall distribution (De Almeida and Valle, 2008). Genotypes capable of maintaining reproductive stability under suboptimal conditions are therefore considered more desirable. The consistent performance of VTLCH2 indicated a greater buffering capacity against environmental stress, allowing it to sustain yield even under unfavorable climatic scenarios, as reported earlier in cocoa and other perennial crops (Asna et al., 2014; Mustiga et al., 2018). Similar adaptive patterns were documented in cashew, mango and wheat, where genotypes with lower environmental responsiveness were recommended for marginal or stress-prone conditions (Sethi et al., 2020; Krishna et al., 2022; Suresh et al., 2025). The adaptability of VTLCH2 therefore aligned with the broader stability principles observed across perennial and annual crops. The similar with findings of (Rahadi et al., 2013) with lowest ranked one were stable in capsicum. Variance-based stability parameters Wricke’s ecovalence (Wi^2^), Ecovalence percentage (Ecov_perc) and Shukla’s variance (σ^2^_i_) and non- parametric in rice was reported by (Aswidinnoor et al., 2023).

In addition to yield stability, biochemical traits such as fat content are influenced by GEIs, with stable genotypes maintaining consistent quality attributes despite environmental variations (Mustiga et al., 2019; Sari et al., 2022). The ability of VTLCH2 to retain a stable biochemical profile suggested efficient metabolic regulation, which was particularly advantageous under low-input or stress-prone environments. Comparable trends were observed in oil palm, where genotypes exhibiting stability under marginal conditions were preferred for sustainable production systems (Hayati et al., 2004; Rafii et al., 2012; M'Bo et al., 2022).

The multivariate stability analyses, including AMMI and related interaction models, provided further insights into the adaptive behavior of the VTLCH2 genotype across environments. Genotypes negatively positioned on PC1 performed well under stress conditions, which often displayed specific adaptation patterns that minimized the GEI effects and near to zero results in reliable or stable yield performance (Gauch, 2013; Susilo, 2018; Tiwari et al., 2018). Similar interpretations were reported in cocoa and oilseed crops, emphasizing the importance of selecting genotypes with predictable performance rather than maximum responsiveness alone (Agahi et al., 2020; Afandi et al., 2023).

Overall, the superiority of VTLCH2 is explained by its strong genetic control over reproductive efficiency, effective assimilate partitioning, physiological resilience to environmental stress, and stable expression of quality traits. These attributes collectively supported its recommendation as a climate-resilient cocoa clone suitable for cultivation under variable and increasingly challenging agro-climatic conditions, particularly in the humid tropics of India.

## Conclusion

The combined regression and AMMI variance-based stability analyses demonstrated VTLCH2 as the most productive cocoa clone, which exhibited superior pod load, higher bean number, and stable fat content across environments. Its *β_i_*< 1, low *S*^2^*d_i_*, and favorable PC1 positioning indicated specific adaptability to marginal or stress-prone environments, making it suitable for humid tropics under climate change conditions. VTLCH4 showed high yield potential, but greater environmental sensitivity, reflecting strong GEI effects. VTLCH1 recorded comparatively low yield and showed the highest stability across yield components. Overall, VTLCH2 emerged as the most promising clone, combining high yield with adaptability to diverse cocoa-growing environments in India. Further research is needed to identify specific physiological events such as bean formation and development on the temperature and water availability, fat content, and fatty acid profile of cocoa.

## Data Availability

The original contributions presented in this study are included in the article/**Supplementary Material**. Further inquiries can be directed to the corresponding author.
