# Supplementary material for: Stability analysis of cocoa clones for yield in the humid tropics of India
Source: Front Plant Sci. 2026 Apr 13;17:1767516. doi: 10.3389/fpls.2026.1767516 (PMC13111314; doi:10.3389/fpls.2026.1767516)
Supplement: Supplementary file 1 [file DataSheet1.pdf]

Supplementary Data

Table . Performance of cocoa clones for growth parameters:

Cocoa clones	Plant height (m)	Girth (m)	Height at 1st branching (m)	Canopy spread	
				E-W (m)	N-S (m)
VTLCC – 1	3.26	0.42	2.43	3.74	3.41
VTLCH – 1	3.09	0.45	2.34	3.95	3.51
VTLCH – 2	3.13	0.42	2.47	3.68	3.33
VTLCH – 3	3.03	0.43	2.42	4.12	3.61
VTLCH – 4	3.17	0.43	2.55	3.72	3.24
VTLC-1	3.39	0.46	2.91	4.27	3.55
S Em ±	0.22	0.01	0.17	0.22	0.17
CD at 5%	NS	NS	NS	NS	NS
CV (%)	13.60	5.03	13.80	11.39	9.72

Layout of experimental design

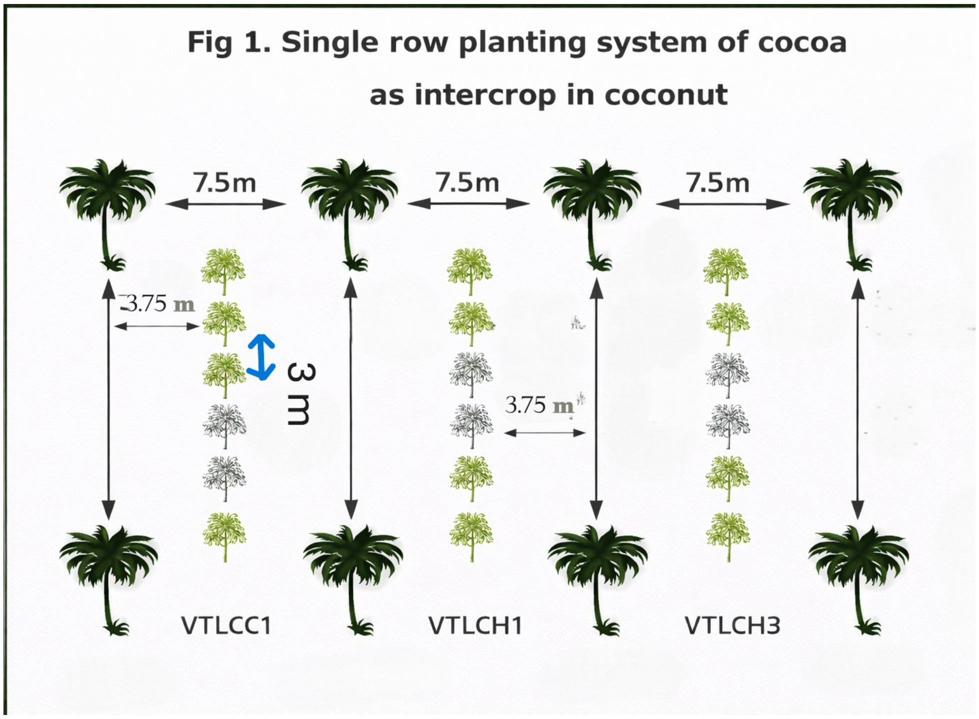

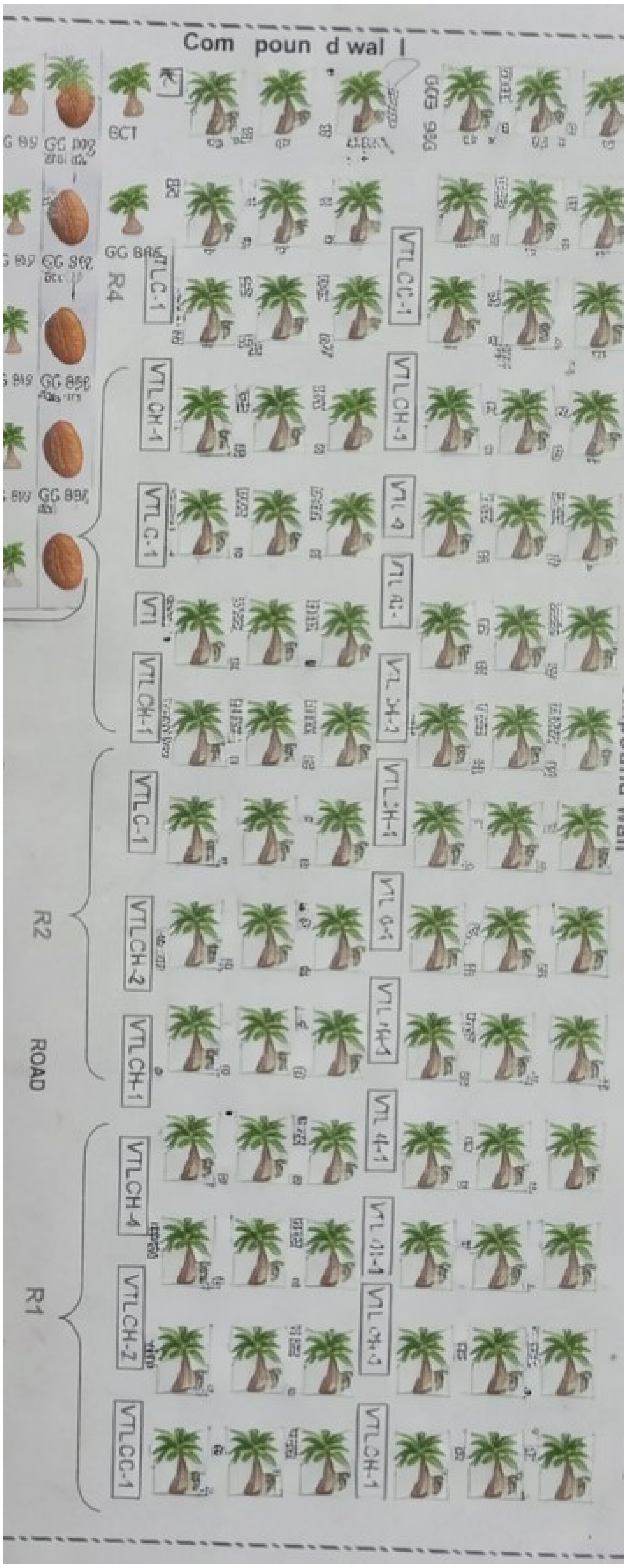
Com poun d wal l
BCT
R4
VTLC-1
VTLCC-1
VTLCH-1
R2
VTLCH-2
ROAD
R1
VTLCH-4
VTLCC-1
